# Downregulation of TGF-β Receptor-2 Expression and Signaling through Inhibition of Na/K-ATPase

**DOI:** 10.1371/journal.pone.0168363

**Published:** 2016-12-22

**Authors:** Jennifer La, Eleanor Reed, Lan Chan, Larisa V. Smolyaninova, Olga A. Akomova, Gökhan M. Mutlu, Sergei N. Orlov, Nickolai O. Dulin

**Affiliations:** 1 Department of Medicine, Section of Pulmonary and Critical Care Medicine, the University of Chicago, Chicago, IL, United States of America; 2 Laboratory of Biomembranes, Department of Biophysics, Faculty of Biology, Lomonosov Moscow State University, Moscow, Russia; 3 Siberian Medical State University, Tomsk, Russia; University of Bergen, NORWAY

## Abstract

Transforming growth factor-beta (TGF-β) is a multi-functional cytokine implicated in the control of cell growth and differentiation. TGF-β signals through a complex of TGF-β receptors 1 and 2 (TGFβR1 and TGFβR2) that phosphorylate and activate Smad2/3 transcription factors driving transcription of the Smad-target genes. The Na^+^/K^+^-ATPase is an integral plasma membrane protein critical for maintaining the electro-chemical gradient of Na^+^ and K^+^ in the cell. We found that inhibition of the Na^+^/K^+^ ATPase by ouabain results in a dramatic decrease in the expression of TGFβR2 in human lung fibrobalsts (HLF) at the mRNA and protein levels. This was accompanied by inhibition of TGF-β-induced Smad phosphorylation and the expression of TGF-β target genes, such as fibronectin and smooth muscle alpha-actin. Inhibition of Na^+^/K^+^ ATPase by an alternative approach (removal of extracellular potassium) had a similar effect in HLF. Finally, treatment of lung alveolar epithelial cells (A549) with ouabain also resulted in the downregulation of TGFβR2, the inhibition of TGF-β-induced Smad phosphorylation and of the expression of mesenchymal markers, vimentin and fibronectin. Together, these data demonstrate a critical role of Na^+^/K^+^-ATPase in the control of TGFβR2 expression, TGF-β signaling and cell responses to TGF-β.

## Introduction

Transforming growth factor-β (TGF-β) is a multi-functional cytokine implicated in regulation of epithelial cell growth [1–3], differentiation of smooth muscle cells [4], myofibroblast transformation [5–7], epithelial-to-mesenchymal transition [8,9] and other cellular processes. TGF-β signals through a receptor kinase complex, consisting of TGF-β receptor type I (TGFβR1) and TGF-β receptor type II (TGFβR2). Upon binding of TGF-β to the receptor complex, TGFβR2 phosphorylates and activates TGFβR1, which in turn phosphorylates SMAD2/3 transcription factors. The phosphorylated SMAD2/3 heterodimerize with SMAD4, translocate to the nucleus and bind to SMAD binding elements (SBE) in target genes to initiate SMAD-dependent gene transcription [10].

The TGF-β signaling pathway is a highly regulated process. There is evidence to support internalization of the TGF-β receptors via clathrin coated pits or lipids rafts plays a role in modulating TGF-β-induced signaling. Endocytosis of TGF-β receptors by clathrin-coated pits to phosphatidylinositol-3 phosphate enriched early endosome, allows for the recruitment of SARA (the SMAD anchor for receptor activation) via the FYVE domain, to mediate Smad phosphorylation [11]. Blocking of clathrin-mediated endocytosis is sufficient to inhibit TGF-β signaling [12]. On the other hand, endocytosis by lipid rafts is associated with decreased signaling by increasing TGF-β receptor degradation. Disruption of lipid rafts by nystatin decreased receptor turnover and therefore enhanced TGF-β signaling [13]. Thus, lipid rafts may play a dual role in TGF-β receptor signaling and receptor downregulation.

The Na^+^/K^+^ ATPase (sodium pump) is an integral plasma membrane protein required for maintaining the electro-chemical gradient of Na^+^ and K^+^ in the cell. The pump is made up of the catalytic alpha subunit and the regulatory beta subunit. The alpha subunit hydrolyzes ATP to pump 3Na^+^ ions out of the cell and 2K^+^ ions into the cell against their concentration gradient. The beta subunit stabilizes the enzyme [14–16] and acts as a molecular chaperone to assist in the transport and insertion of the alpha subunit to the plasma membrane [17]. A family of drugs known as cardiac glycosides, including digoxin and ouabain, bind to the catalytic alpha subunit and are pharmacological inhibitors of the Na^+^/K^+^ ATPase [18]. Digoxin, isolated from Digitalis lanata [19], has been used for treatment of congestive heart failure and cardiac arrhythmias [20]. Ouabain, isolated from Strophanthus gratus [21], is the most commonly used cardiac glycoside for *in vitro* studies due to its high water solubility. Inhibition of Na^+^/K^+^ ATPase by cardiac glycosides leads to an increase in the intracellular Na^+^/K^+^ ratio and depolarization of cells, resulting in the activation of reverse mode of Na^+^/Ca^2+^ exchanger and of voltage-gated Ca^2+^ channels, respectively, both leading to an increase in the intracellular Ca^2+^ concentrations [22].

Recent studies have suggested that cardiac glycosides, through binding to Na^+^/K^+^-ATPase, can also affect cell growth signaling pathways. It was shown initially that ouabain and marinobufagenin induced proliferation of vascular smooth muscle cells [23]. Subsequently, it was demonstrated that ouabain stimulated Src / epidermal growth factor receptor (EGFR) signaling leading to the activation of extracellular signal regulated proteins kinases (ERK1/2) and of phosphatidylinositol-3-kinase (PI3K) in various cell types [24–26]. It was reported that at least some of these effects of ouabain (i.e. activation of ERK1/2, but not of PI3K) are mediated through a direct interaction and activation of Src by ouabain-bound Na^+^/K^+^ ATPase [27–29]. Interestingly, the signaling role of Na^+^/K^+^ ATPase was reported to be associated with caveolae, where significant amounts of Src, EGFR, ERK1/2 and α1/2-subuntis of Na^+^/K^+^-ATPase have been detected [30,31]. However, this signaling role of caveolae seems to be dissociated from the Na^+^/K^+^ pump activity and from regulation of cardiac contractility by Na^+^/K^+^ ATPase [32–34].

In this study, we show for the first time that inhibition of the Na^+^/K^+^ ATPase leads to substantial downregulation of TGFβR2 expression at mRNA and protein levels resulting in the inhibition of TGF-β signaling and cellular responses, and this effect is likely mediated through inhibition of the Na^+^/K^+^ pump activity of Na^+^/K^+^-ATPase.

## Materials and Methods

### Primary Culture of Human Lung Fibroblasts

Human lung fibroblasts were cultured as described previously [6,7]. Briefly, human lung parenchyma was minced to ∼1-mm^3^ pieces, washed, and plated on 10-cm plates in growth medium containing Dulbecco’s modified Eagle’s medium (DMEM) supplemented with 10% FBS, L-glutamine, and antibiotics. The medium was changed every two days. After ∼2 weeks, the explanted and amplified fibroblasts were cleared from the tissue pieces, trypsinized, and further amplified as passage 1. For experiments, cells were grown in 12-well plates at a density of 1 × 10^5^ cells per well in growth medium for 24 hours, starved in DMEM containing sterile bovine serum albumin at 0.1% (DMEM/BSA) for 24 hours, and treated with desired drugs or with DMEM/BSA containing or lacking potassium chloride (5mM) for desired times.

### Reverse Transcription-quantitative Real-time PCR

RNA STAT-60 (TEL-Test) was used to isolate total RNA following the manufacturer's protocol. RNA was randomly primed and reverse transcribed using the iScript cDNA synthesis kit (Bio-Rad, Hercules, USA) according to the manufacturer's protocols. Real-time PCR analysis was performed using iTaq SYBR Green Supermix with ROX (Bio-Rad) in a MyIQ single-color real-time PCR detection system (Bio-Rad), The TGFΒR2 primers were: GGAGTTTCCTGTTTCCCCCG (forward) and ATGTCTCAGTGGATGGGCAG (reverse). The 18s primers were: GATTAAGTCCCTGCCCTTTG (forward) and GTTCACCTACGGAAACCTTG (reverse).

### Western Blotting

Cells were lysed in urea buffer containing 8 M deionized urea, 1% SDS, 10% glycerol, 60mM Tris-HCl pH 6.8, 0.02% pyronin Y, and 5% β-mercaptoethanol. Lysates were sonicated for 5 s. Samples were then subjected to polyacrylamide gel electrophoresis and Western blotting with desired primary antibodies and corresponding horseradish peroxidase (HRP)-conjugated secondary antibodies, and developed by chemiluminescence reaction (Pierce). Digital chemiluminescent images below the saturation level were obtained with a LAS-4000 analyzer, and the light intensity was quantified using Multi Gauge software (Fujifilm).

### Reagents

Dulbecco’s Modified Eagle Medium was from Thermofisher, catalog number 11960044. L-glutamine was from Thermofisher, catalog # 25030081. Antibiotic-Antimycotic was from Thermofisher, catalog # 15240062. Ouabain octahydrate was from Sigma-Aldrich, CAS # 11018-89-6. TGF-β was from EMD Millipore, catalog # GF111 (Billerica, MA). Pharmaceutical grade bleomycin (BLEOmycin) was from TEVA (lot#: 31314497B). iScript cDNA Synthesis (catalog # 1708891) and iTaq SYBR Green Supermix (catalog # 1725121) were from Bio-Rad. Antibodies for Western blotting against SM α-actin (catalog # A5228-200ul), β-actin (catalog # A5441-.2ML), and α-tubulin (catalog # T6074-200ul) were from Sigma-Aldrich; collagen-1 was from Cedarlane laboratories (catalog # CL50111AP-1), and transforming growth factor-beta type 2 receptor antibodies were from Santa Cruz (catalog # SC-400)

### Statistical Analysis

Quantitative data from three independent experiments were analyzed by Student's T-test. Values of p < 0.05 were considered statistically significant.

## Results

### Inhibition of the Na^+^/K^+^ ATPase Downregulates the mRNA and Protein Expression of TGFβR2

**Fig 1:**
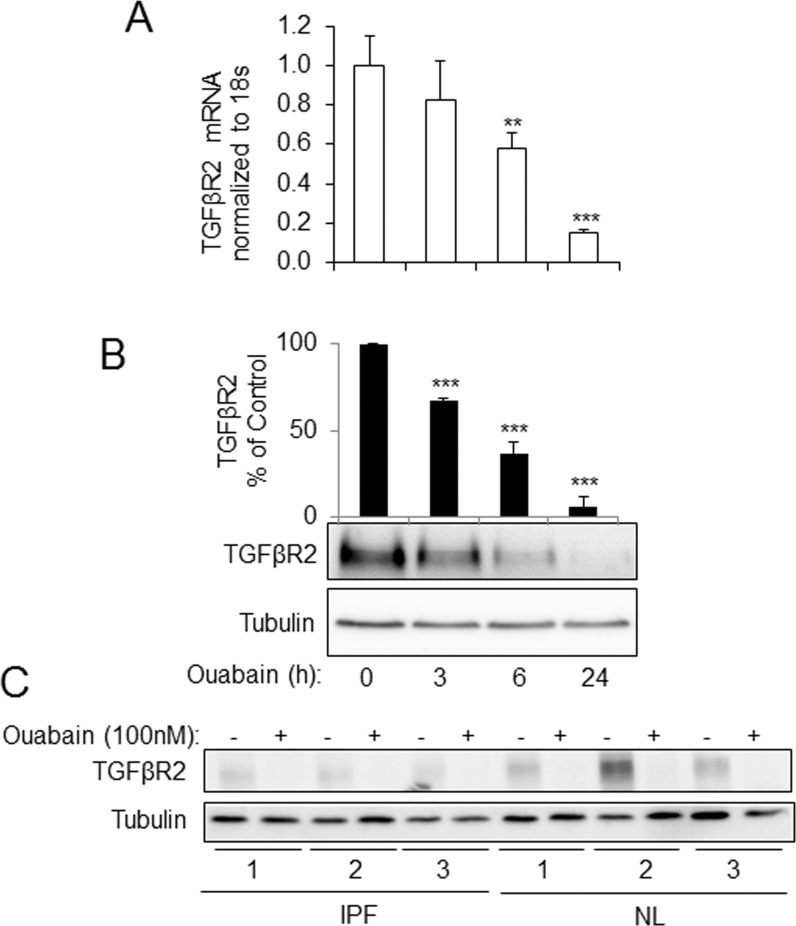
Ouabain down-regulates TGFβR2 mRNA and protein levels in human lung fibroblasts (HLF). Serum-starved HLF were treated with 100 nM ouabain for 3, 6, and 24 hours. Cells were analyzed by real-time qPCR for TGFβR2 mRNA levels (**A**) and by Western blotting for TGFβR2 protein levels (**B**). Shown are the representative images and the quantitative analysis of ECL (mean±SD) from at least three independent experiments (RLU, relative light units). **p < 0.005, ***p < 0.0005. **C.** HLF isolated and cultured from IPF and non-IPF (NL) lungs were treated with or without 100 nM ouabain. Cells lysates were analyzed for TGFβR2 or tubulin protein expression by Western blotting.

**Fig 2:**
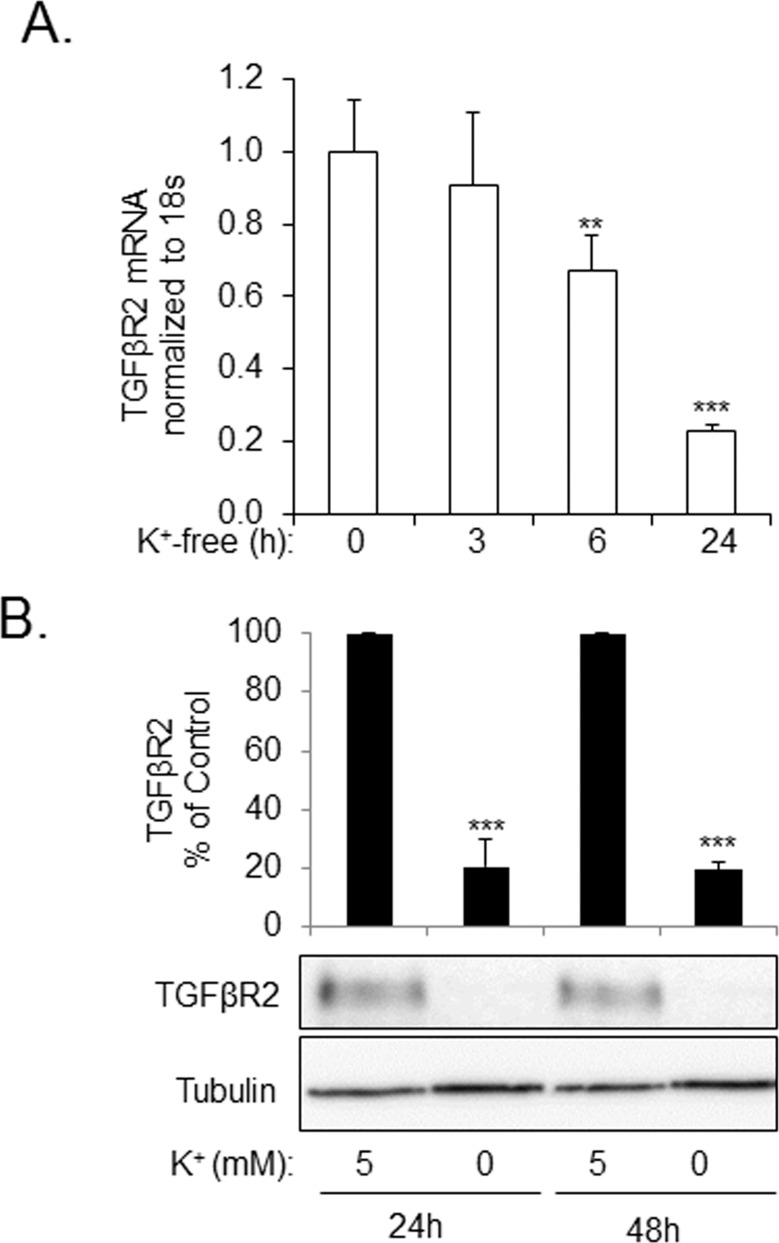
Inhibition of Na^+^/K^+^ ATPase by potassium free media results in the down-regulation of TGFβR2 mRNA and protein levels in human lung fibroblasts (HLF). **A**. HLFs were placed in potassium free media for 3, 6, and 24 hours, and then analyzed for TGFβR2 mRNA levels via real time qPCR. **B**. HLFs were treated with media containing either 5 mM KCl or 0 mM KCl for 24 or 48 hours. Cell extracts were examined by Western blotting for TGFβR2 expression or tubulin. Shown are the representative images and the quantitative analysis of ECL (mean±SD) from at least three independent experiments (RLU, relative light units). **p < 0.005, ***p < 0.0005.

Fig 1 shows that treatment of human lung fibroblasts (HLFs) with 100 nM ouabain resulted in a time-dependent downregulation of TGFβR2 mRNA (Fig 1A) and protein (Fig 1B) levels, as assessed by real time qPCR and Western blotting, respectively. The inhibitory effect of ouabain on TGFβR2 expression was further examined using primary HLF cultures isolated from lungs of six individuals—three with idiopathic pulmonary fibrosis (IPF) and three with non-fibrotic lungs (NL). As shown in Fig 1C, while variable expression of TGFβR2 between the HLF cultures was observed, 100 nM ouabain substantially reduced TGFβR2 expression in all six HLF cultures. In order to determine whether the downregulation of TGFβR2 by ouabain was due to the inhibition Na^+^/K^+^ ATPase, we used an alternative approach for inhibition of Na^+^/K^+^ ATPase through a removal of extracellular potassium. As shown in Fig 2, incubation of HLFs in a potassium-free medium resulted in a sustained inhibition of the expression of TGFβR2 mRNA and protein as compared to the medium containing 5 mM KCl.

### Downregulation of TGFβR2 Through Inhibition of the Na^+^/K^+^-ATPase Results in Decreased Cellular Responses to TGF-β

**Fig 3:**
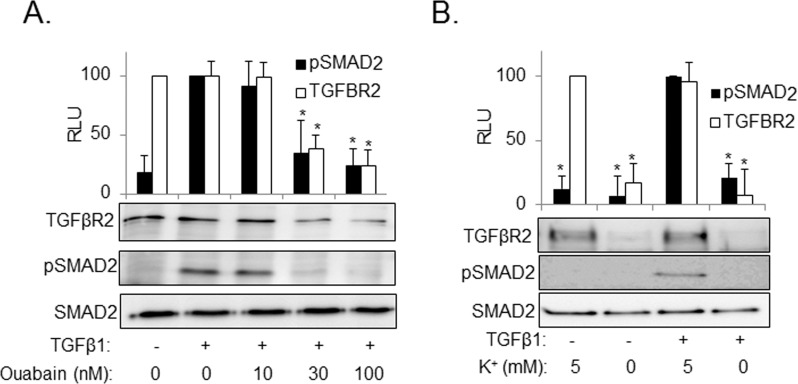
Inhibition of the Na,K-ATPase by potassium free media or by ouabain blocks TGFβ1-induced SMAD2 phosphorylation. HLF were pretreated with ouabain at 10, 30,100 nM (**A**) or placed in media containing 5 mM KCl or 0 mM KCl (**B**) for 24 hours. Cells were then stimulated with 1ng/ml of TGFβ1 for 1 hour. Cell lysates were analyzed by Western blotting with desired antibodies as indicated. Shown are the representative images and the quantitative analysis of ECL (mean±SD) from at least three independent experiments (RLU, relative light units). *, *p* < 0.05.

**Fig 4:**
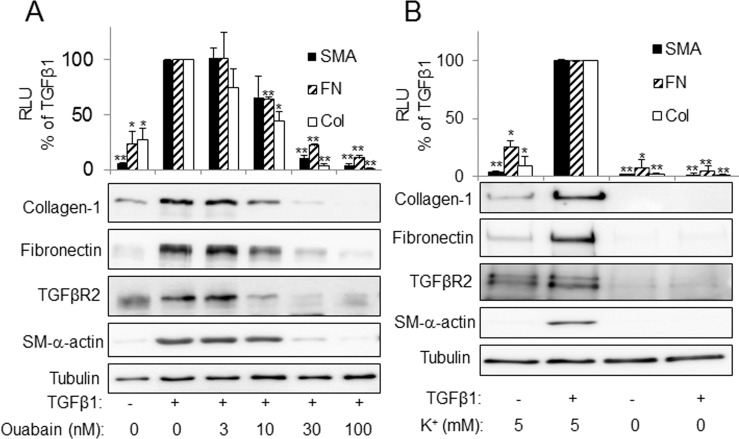
Inhibition of the Na,K-ATPase by potassium free media and ouabain blocks TGFβ1-induced myofibroblast differentiation. HLF were treated with increasing doses of ouabain (**A**) or placed in media containing 5 mM KCl or 0 mM KCl (**B**) and stimulated with or without 1 ng/ml of TGFβ1 for 24 hours. Cell lysates were then analyzed by Western blotting with desired antibodies as indicated. Shown are the representative images and the quantitative analysis of ECL (mean±SD) from at least three independent experiments (RLU, relative light units). *p < 0.005, **p < 0.0005.

To determine whether downregulation of TGFβR2 through inhibition of Na^+^/K^+^-ATPase translates to the functional cell responses to TGF-β, we treated HLFs with TGF-β in the presence or absence of ouabain. As shown in Fig 3, TGF-β-induced phosphorylation of Smad2 was inhibited by ouabain (Fig 3A) or through incubation in potassium-free media (Fig 3B). Furthermore, ouabain or removal of extracellular potassium blocked TGF-β-induced expression of TGF-β-target genes such as fibronectin or α-smooth muscle actin (α-SMA) (Fig 4), without affecting the levels of tubulin or caveolin-1 (S1 Fig).

**Fig 5:**
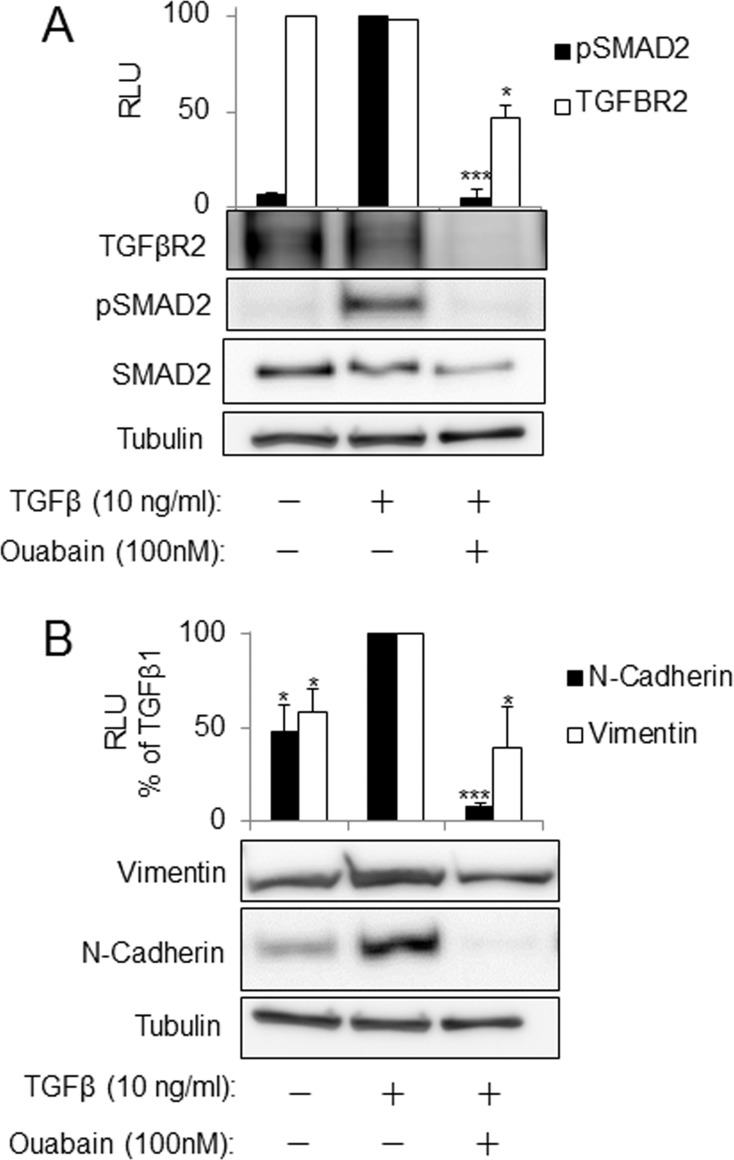
Downregulation of TGFβR2 by ouabain is accompanied by inhibition of TGFβ1-induced SMAD2 phosphorylation and of the expression of mesenchymal markers in A549 cells. Serum starved A549 cells were pretreated with increasing doses of ouabain for 24 hours followed by stimulation with 10ng/ml of TGFβ1 for 1 hour (**A**) or simultaneously treated with ouabain and TGFβ1 for 48 hours (**B**). Cell lysates were then analyzed by Western blotting with desired antibodies as indicated. Shown are the representative images and the quantitative analysis of ECL (mean±SD) from at least three independent experiments (RLU, relative light units). *, *p* < 0.05, **p < 0.005, ***p < 0.0005.

To determine whether the inhibitory effects of ouabain on TGFβR2 expression that we observed in fibroblasts could be generalized to other cells, we treated human alveolar epithelial cells (A549) with TGF-β in the presence and absence of ouabain. Fig 5 demonstrates that treatment of A549 cells with ouabain inhibits TGFβR2 expression, TGF-β-induced Smad2 phosphorylation and the expression of TGF-β target genes, vimentin and N-cadherin.

## Discussion

Cardiac glycosides have been used extensively for the treatment of cardiac arrhythmias and heart failure due to their positive inotropic effects. Inhibition of the Na^+^/K^+^ ATPase and the resultant increase in intracellular Ca^2+^ concentration in cardiac myocytes is considered a major mechanism for the therapeutic effects of cardiac glycosides. Our study for the first time demonstrates that inhibition of Na^+^/K^+^ ATPase by two independent approaches (ouabain and K^+^-free media) downregulates TGFβR2 mRNA and protein levels in human lung fibroblasts and alveolar epithelial (A549) cells, resulting in the loss of TGFβ-induced signaling (SMAD2 phosphorylation) and functional responses to TGF-β (myofibroblast differentiation and EMT).

At this time, the precise mechanism by which ouabain downregulates the expression of TGFβR2 is not clear. It is well established that binding of ouabain to Na^+^/K^+^ ATPase results in the inhibition of its pump activity. In our experiments, the depletion of extracellular potassium, an alternative approach of inhibiting the Na^+^/K^+^ ATPase, resulted in effects similar to ouabain treatment on TGFβR2 expression, TGF-β-induced Smad2 phosphorylation and gene expression. This suggests that the effects of ouabain are likely mediated by inhibition of the Na^+^/K^+^ -ATPase pump activity. On the other hand, increasing evidence suggests that cardiac glycosides may promote cell growth signaling through Src, EGFR, ERK and PI3K in caveolae [23–31]. Even though these caveolae-mediated signaling events may be dissociated from the pump activity of Na^+^/K^+^ ATPase [32–34], it is possible that these signaling events may also contribute to the downregulation of TGFβR2 expression by ouabain. Understanding the mechanism by which cardiac glycosides downregulate TGFβR2 expression is our current goal.

Myofibroblast differentiation and possibly EMT are thought to represent critical processes in wound healing and pathogenesis of fibrosis during congestive heart failure [35–37], secondary or idiopathic pulmonary fibrosis [38–41], advanced kidney disease [42–44], scleroderma [45,46], and liver cirrhosis [47,48]. Importantly, TGF-β levels are upregulated in fibrotic organs; and TGF-β signaling is thought to be critical for the development of fibrosis [49,50]. Thus, it is conceivable that if cardiac glycosides decrease the expression of TGFβR2 and TGF-β signaling *in vivo* they may have potential as anti-fibrotic agents.

In addition to treatment of heart disease, recent studies may suggest that cardiac glycosides may be re-purposed for treatment of other diseases, such as cancer [51,52]. The first observation of possible anti-cancer effects of cardiac glycosides was noted in 1979 by Dr. Stenkvist [53]. He noticed that tumor cells in breast cancer patients taking cardiac glycosides showed more benign characteristics as compared to patients not taking cardiac glycosides [53]. Moreover, patients with breast cancer taking digitalis were ~10 times less likely to have recurring cancer within 5 years after mastectomy, suggesting that cardiac glycosides may play a role in modulating aggressiveness of the tumor [54]. A 20-year follow-up study showed that 6% as compared to 34% patients died from breast cancer when on digitalis compared to those not on digitalis [55]. Given our new data and the reported role of TGF-β in stroma development and metastasis [56–58], it is plausible that cardiac glycosides may have anti-cancer activity through downregulation of TGFβR2 in relevant cells. However, while multiple studies have demonstrated the anti-proliferative [24,59] and pro-apoptotic effects [60,61] of cardiac glycosides on various cancer cells *in vitro*, the effect of cardiac glycosides in the *in vivo* models of cancer has not been rigorously assessed.

On the other hand, our findings may also warn about potential adverse effects of cardiac glycosides (in addition to overall toxicity). TGF-β signaling is required for efficient wound healing [62,63]; hence, if cardiac glycosides attenuate the expression of TGFβR2 *in vivo*, one would predict that cardiac glycosides would be harmful during the external or internal injury. Further, it is now established that TGF-β regulates T cell function through driving differentiation of regulatory Treg cells and inhibition of T-helper cells [64,65]. Thus, inhibition of TGF-β signaling by cardiac glycosides may potentially lead to increased T cell responses and enhanced inflammation.

The caveat in addressing the discussed above potential benefits and limitations of cardiac glycosides is behind the fact that in rodents (which are largely used as animal models of disease in experimental research), α1 subunit of Na^+^/K^+^-ATPase is insensitive to therapeutic doses of cardiac glycosides [66]. Therefore, wild type rodents may not be used for assessing the therapeutic effect of cardiac glycosides in organs/cells expressing α1-Na^+^/K^+^-ATPase. The resistance to cardiac glycosides is caused by a substitution of Gln111 and Asn122 of human α1-subunit for Arg and Asp, respectively, in rodents. Based on this, Lingrel and co-workers have generated a mouse with knock-in of ouabain-sensitive α1 isoform of Na^+^/K^+^-ATPase and have used it to delineate the role of α1 in blood pressure regulation, cardiac and skeletal muscle contraction and renal salt handling [67].

### Ethical Aspects of the Proposed Research

#### Human subjects

This study largely used primary cultures of human pulmonary fibroblasts (HPF) established from human lungs rejected for transplantation through the Regional Organ Bank of Illinois (ROBI) / Gift of Hope Network. No IRB protocol is required for using ROBI lungs from deceased individuals. The IPF-HLF were isolated and cultured from lungs of de-identified IPF patients shortly after lung transplantation at the University of Chicago under the IRB protocol #14514A.

## Supporting Information

S1 Fig Ouabain does not effect Caveolin-1 expression in HLF. Serum starved HLF were treated with or without 1 ng / ml TGFβ1 and / or 30 nM ouabain for 24 hours. Cells were lysed and analyzed by Western blotting with desired antibodies.
(PPTX)
