# Supplementary material for: Downregulation of TGF-β Receptor-2 Expression and Signaling through Inhibition of Na/K-ATPase
Source: PLoS One. 2016 Dec 22;11(12):e0168363. doi: 10.1371/journal.pone.0168363 (PMC5179089; doi:10.1371/journal.pone.0168363)
Supplement: S1 Fig — Serum starved HLF were treated with or without 1 ng / ml TGFβ1 and / or 30 nM ouabain for 24 hours. Cells were lysed and analyzed by Western blotting with desired antibodies. (PPTX) [file pone.0168363.s001.pptx]

## Slide 1
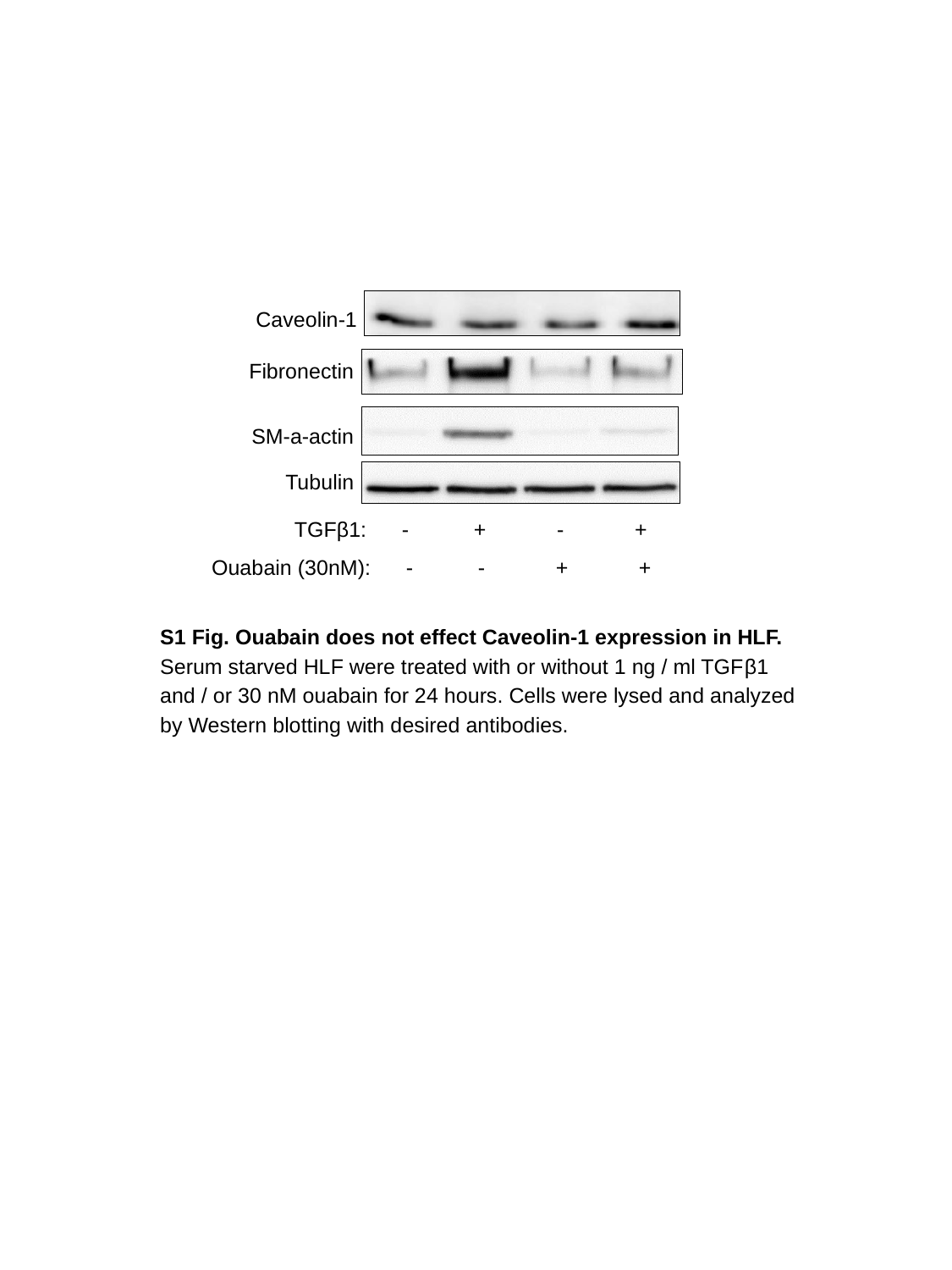

Caveolin-1
Fibronectin
SM-a-actin
Tubulin
TGFβ1: - + - +
Ouabain (30nM): - - + +
S1 Fig. Ouabain does not effect Caveolin-1 expression in HLF.
Serum starved HLF were treated with or without 1 ng / ml TGFβ1 and / or 30 nM ouabain for 24 hours. Cells were lysed and analyzed by Western blotting with desired antibodies.
